# Feces DNA analyses track the rehabilitation of a free-ranging beluga whale

**DOI:** 10.1038/s41598-022-09285-8

**Published:** 2022-04-19

**Authors:** Babett Günther, Eve Jourdain, Lindsay Rubincam, Richard Karoliussen, Sam L. Cox, Sophie Arnaud Haond

**Affiliations:** 1 ISEM (Institut des Sciences de l′Evolution), Univ Montpellier, CNRS, EPHE, IRD, Montpellier, France; 2 MARBEC (Marine Biodiversity Exploitation and Conservation), Univ Montpellier, CNRS, Ifremer, IRD, Sète, France; 3 Norwegian Orca Survey, Andenes, Norway; 4 MaREI Centre, Environmental Research Institute, University College Cork, Cork, Ireland; 5 School of Biological, Earth, and Environmental Sciences, University College Cork, Cork, Ireland

**Keywords:** Ecology, Environmental sciences, Ecology, Behavioural ecology, Biodiversity, Conservation biology, Molecular ecology, Restoration ecology

## Abstract

Following the sudden appearance, and subsequent efforts to support the survival of a beluga whale (*Delphinapterus leucas*) speculated to have been previously trained off the coast of Norway, we investigate the animal’s ability to readapt to life in the wild. Dietary DNA (dDNA) analysis was used to assess diet throughout this rehabilitation process, and during a return to unassisted foraging and self-feeding. Metabarcoding of feces collected throughout this process, confirmed the diversification of the beluga whale’s diet to local prey. These findings are indicative of improved foraging behavior, and the ability of this individual to resume wild foraging following a period of dependency in managed care. New insight of digestion rates, and the time window during which prey detection through dDNA analysis is appropriate was also obtained. Beyond the case study presented here, we demonstrate the power of dDNA analysis as a non-intrusive tool to assess the diet of large mammals and track progress adapting to life in the wild following release from captivity and rehabilitation programs.

## Introduction

**Figure 1:**
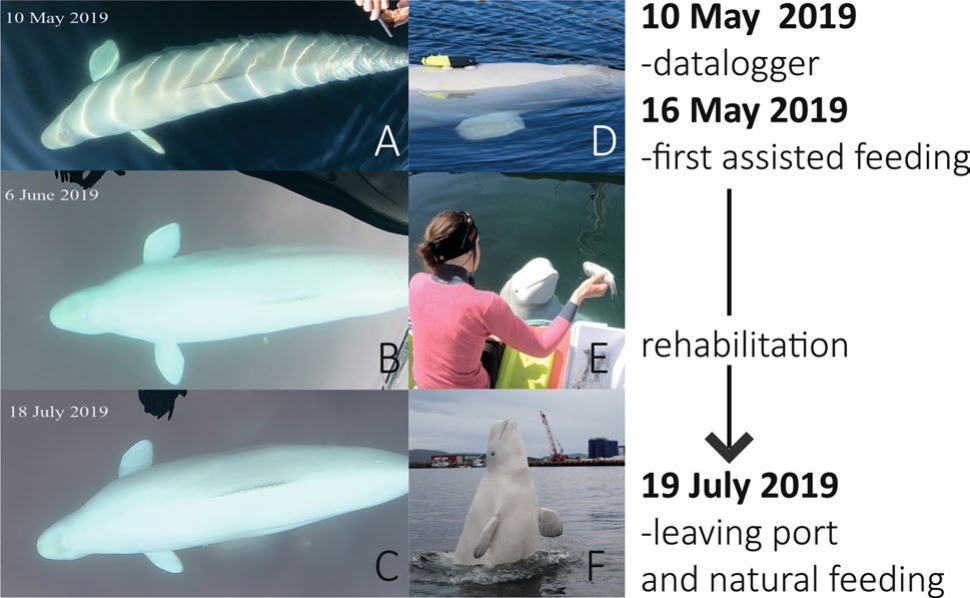
Pictures of beluga whale ‘Hvaldimir’ in summer 2019; (**A**–**C**) Photos show observations of his body condition when at Hammerfest between May and July 2019; (**D**) with the back-mounted, suction cup attached GoPro video camera used to record foraging behavior; (**E**) being hand-fed herring during a routine feeding session; and (**F**) in the harbor of Hammerfest, northern Norway, in July 2019, playful and seeking human contact.

A male beluga whale (*Delphinapterus leucas*), with a harness fitted to his body, was first sighted in Tufjord, northern Norway on 26 April 2019. The harness had an equipment mount attachment, labelled in English “Saint Petersburg Equipment”. Based on these observations and geographic considerations, it was hypothesized that the animal had previously been trained and used in the Russian Navy, though this was never confirmed. On 30th April 2019, the beluga whale appeared in the harbor of Hammerfest where he remained (unrestricted) until mid-July of the same year. While in Hammerfest, the whale quickly engaged with people and triggered substantial interest on social media, rapidly becoming a celebrity in Norway nick-named ‘Hvaldimir’ (a combination of ‘Hval’ for ‘whale’ in Norwegian and ‘-dimir’ in reference to Russian President Vladimir Putin; Fig. 1).

If, as suggested by the harness, the whale originated from a managed-care facility, he had likely been conditioned to be hand-fed. Therefore, concern was raised that the animal may not be receiving the amount of daily nutrition required for long-term survival through self-feeding. To assess the whale’s condition, the research organization Norwegian Orca Survey (NOS) travelled onsite on 7 May 2019. Behavioral observations, i.e., notes taken *ad libitum*^1^ from shore, on the whale’s surface activities and movements within the harbor, were collected for 8–10 h a day between the 7th and 12th May. On the 10th May, the whale was equipped with a datalogger encompassing an inbuilt HD camera and motion sensors (accelerometer, magnetometer and gyroscope; Customized Animal Tracking Solutions, Germany) to gain further insights of his underwater activities (Fig. 1D). The datalogger was attached by hand using suction cups when the whale made contact with the team, with no need to restrain the animal. After 12 h of attachment, the datalogger was retrieved following the same procedure. Behavioral data collected from both the observer (EJ) and the datalogger failed to indicate the presence of feeding activity (E.J., pers. comm.). Notably, the beluga whale spent most of his time logging at the surface by the dock in the inner harbor, or engaging with people. Further assessment, via visual observation of underwater and surface images made using a GoPro camera on a stick, suggested a lean body condition (Fig. 1A,D). Following review by a team of international professionals experienced with the management of beluga whales, NOS proposed to implement a feeding program with the immediate goal of improving Hvaldimir’s condition and survival, hereafter referred to as a ‘rehabilitation process’. An official permit from the Norwegian Directorate of Fisheries was acquired on 16 May 2019 after which NOS initiated an assisted feeding program for Hvaldimir, supported by both local and international sponsorship (Fig. 1C).

From May 16th, the whale was cautiously fed with 5–7 kg of frozen-thawed Atlantic herring (*Clupea harengus*) throughout four to six daily feeding sessions. Gradually, his food intake was increased to 15–20 kg a day. Within just a few days, Hvaldimir displayed higher levels of energy. He spent less time logging by the dock, and started using the entire harbor area (~ 0.7 km^2^). Continued monitoring using a 4 K camera (GoPro Hero 4 Black edition) attached with suction cups (without motion sensors) further revealed the whale attempting to capture live fish on multiple occasions outside of the assisted feeding sessions (E.J. pers. comm.). Following an apparent improvement in physical activity, and after signs of foraging were observed, a reduced and variable daily food-provisioning program was initiated with the hope to encourage natural foraging and Hvaldimir’s autonomy in feeding. In parallel, fecal samples were collected opportunistically for dietary analysis. Over time, based on visual assessment, Hvaldimir’s body condition appeared more robust (Fig. 1A–C), though this could not be quantified because the whale movements during encounters did not allow the team to take detailed measurements of his body.

On 19 July 2019, after 2.5 months of routine assisted feeding, Hvaldimir left Hammerfest and travelled along the Norwegian coast, stopping in many different places where he stayed for various periods of time (E.J, L.R, R.K pers. comm., based on observations from local residents). A brief visit back to Hammerfest was made at the beginning of September. Limited logistics to reach these mostly remote places only allowed for short, intermittent periods of observation for the rest of 2019. Thus, innovative means were required to assess Hvaldimir’s feeding activity during opportunistic rare encounters, and DNA based analysis of feces was thus considered.

On six occasions between June and September 2019 (spanning the initial period of rehabilitation process in Hammerfest and subsequent opportunistic encounters), non-invasive feces collection was used to further extract DNA to infer diet evolution. It was hoped a diversification other than provisioned herring would be detected, which would be a clear indication of active and successful foraging following the rehabilitation process undertaken while Hvaldimir resided in Hammerfest. Metabarcoding combining sequence-based identification with high-throughput sequencing technology (HTS; Pompanon et al.^2^) has developed over the past few years as a powerful and non-intrusive (when applied to feces rather than stomach contents^3^) method to study diet. Its use has recently been extended to marine mammals like sea lions^4^, walruses^5^, and killer whales^6^. Several studies have now indicated that relative read abundance of fecal DNA is representative of stomach content, and offers reliable qualitative and semi quantitative results^3,7–9^. For our study, two gene regions were chosen; mitochondrial cytochrome *c* oxidase subunit I (COI), which allows species-specific detection, especially of fish, and ribosomal 18S which supports the general broad range detection of invertebrates as potential prey^10^.

The rehabilitation or rewilding of marine mammals is a complex, broad, and highly debated topic^11,12^. In general, the release of animals into the wild, that were once dependent on human care (or even captive), requires ascertaining the animal is healthy and has the essential skills for survival. Self-feeding is an obvious necessity, yet is challenging to monitor. Here, we describe produced metabarcodes of the DNA extracted from feces to analyse diet composition through dietary DNA (dDNA). We aim to assess if Hvaldimir’s feeding included wild prey at some point during his monitoring, and secondly if nutrition changes could be detected at various stages of the rehabilitation process.

## Methods

### Sampling and DNA extraction

Feces sampling commenced with the onset of Hvaldimir’s assisted feeding (Table 1, Fig. 1E). Samples were then collected opportunistically throughout summer 2019, during land-based feeding or husbandry sessions. Initially, samples were collected from the dock, as the animal spontaneously defecated while standing vertically with his head out of the water. Fecal pellets were collected manually, within a few seconds of defecation, when they reached the sea surface and floated. Sampling procedure was the same in Altneset where interactions with the whale happened from a 4 m fiber glass boat. Six fecal samples (a sample describes all material from one defecation) were collected between June and September 2019 (Table 1). Upon collection, samples were stored within separate plastic vials (falcon tubes or large eppendorfs). They were then transported (frozen at − 20 °C) to a laboratory in France (Ifremer-MARBEC, Sète) for DNA extraction.

**Table 1:** Information related to sampling of Hvaldimir’s fecal pellets between June and September 2019 in northern Norway.

Sample id	Stage	Date	Time	Location	Notes
#1	Day 24	8 Jun	15:45	Hammerfest harbor	Time since last feed (5 kg herring) was ~ 4 h
#2	N/A	Jun–Jul	N/A	Hammerfest harbor	No exact date/time; was collected in daytime between two herring feeds
#3	Day 92	15 Aug	evening	Altneset, Seiland	Collected during a six-week period when Hvaldimir was not provisioned with food
#4	Day 110	2 Sept	13:45	Hammerfest harbor	Collected after Hvaldimir had returned to Hammerfest harbor (on 30th Aug) following six weeks spent on his own; time since last feed (5 kg herring) was ~ 1h40 min
#5	Day 111	3 Sept	17:45	Hammerfest harbor	Time since last feed (4.5 kg herring) was 7.5 h
#6	Day 112	4 Sept	09:45	Hammerfest harbor	Time since last feed (4.4 kg herring) was 15 h (night before)
PC		–	–	–	Positive control: Atlantic herring (*Clupea harengus*)

From each sample, the whole material collected from a defecation event (about ~ 5–10 g of feces was used for DNA extraction. DNA extraction was performed using a PowerMax Soil DNA Isolation Kit (Qiagen, Hilden, Germany) to maximize the amount of starting material. Due to a potential protein content in such samples not observed in ones from soil, a first lyses step was included using 750 µl bead solution, 65 µl of solution C1 and 20 µl of proteinase K incubated for 30 min 65 °C. All other steps were performed following manufacturer’s instructions. Two controls, one positive (herring flesh) and one negative (an empty DNA extraction column), were added for quality control of the bioinformatics processes (decontamination and tag switching, see below). This followed the exact same steps as the matched extraction batch.

For all DNA samples and controls, PCR was performed separately for two barcoding gene regions: (1) COI (Metazoa) using mlCOIintF GGWACWGGWTGAACWGTWTAYCCYCC^13^, andjgHCO2198 TANACYTCNGGRTGNCCRAARAAYCA^14^ for an approximate fragment length of 313 bp, and (2) 18S-V1V2 (Metazoa) with SSUF04 GCTTGTCTCAAAGATTAAGCC^15^, and SSURmod CCTGCTGCCTTCCTTRGA^16^, for a variable ribosomal loop length of 300 to 500 bp. Compared to these original primer sequences, Inosine (I) was changed with “wobbles” (N) to create degenerate primers compatible with the High-Fidelity Phusion Taq Polymerase used (which does not otherwise recognize Inosine). All primers were synthesized with standardized Illumina adapters: forward TCGTCGGCAGCGTCAGATGTGTATAAGAGACAGMKGGWACW, and reverse GTCTCGTGGGCTCGGAGATGTGTATAAGAGACAGMK, and later combined with Illumina barcodes to allow multiplexing. Each 30 µl amplification reaction contained 2 µl of DNA template, 0.7 pM of each primer, and 15 µl of Phusion® High-Fidelity PCR 2X Master Mix with GC buffer (New England Biolabs, Ipswich, MA US), with the remaining volume made up of molecular water. Additionally, all COI reactions included an additional 1 mM MgCl_2_ (1.2 µl/25 mM), which together with the 1.5 mM MgCl_2_ contained in the Mix-Phusion resulted in 2.5 mM MgCl_2_. The PCR cycling conditions were as follows: 98 °C for 30 s, followed by a marker specific number of 10 s cycles (35 for 18S and 40 for COI) at 98 °C, annealing (57 °C for 18S-V1 and 48 °C for COI) for 45 s, and 72 °C for 60 s, with a final elongation at 72 °C for 10 min. All PCR products were examined via gel electrophoreses and quantified using a Qubit 3.0 fluorometer (Invitrogen, Denmark). Sequencing and library preparation were performed by the Labex platform GenSeq (Montpellier University, France). The Nextera XT Index Kit (Illumina, Hayward, CA, USA) was used separately for each gene region for Library prep (PCR based), including PhiX for standardization. Sequencing was performed on an Illumina MiSeq instrument with the corresponding reagent kit (2 × 250 bp).

### Bioinformatics

Data were analyzed following the bioinformatic pipeline described by Brandt et al.^17^. The FASTQ files were first processed using Cudadapt^18^ to remove all primers and leftover adapters. An error correction algorithm was then implemented by the program DADA2^19^, after pre-filtering reads using a maximum expected error (MaxEE) of 5 and truncation length of 250 bp (with minimum truncQ = 2). The parameters used for fragment size selection were an expected total length of 250–350 base pairs (bp) for COI, and 300–500 bp for 18S-V1V2 assembled fragments. A chimaera removal step was included. A list of unique sequences referred to as amplicon sequence variants (ASVs) was obtained along with the number of their occurrences (reads) in each sample and control. To avoid confounding intraspecific diversity and species diversity, particularly for metazoans^17,20^, processed ASVs were clustered into operational taxonomic units (OTUs) using the program swarm2^21^, with an iterative local threshold d of 6 for COI and 4 for 18S-V1V2.

Taxonomic assignment was performed at the ASV level against the reference database Silva release 132^22^ for 18S-V1V2 ribosomal sequences, and Midori^23^ for COI. Assignments were performed using the RDP naive Bayesian classifier method^24^. Possible cross-contaminants introduced during extraction, PCR, and sequencing were removed using the prevalence method (with a threshold of 0.5) from the *decontam*^25^ package in R, which integrates information on the identity and number of reads found in the samples and PCR-negative controls. For all gene regions, the final OTU counts were adjusted using an R-based script^26^ to account for potential tag switches that can occur during library preparation^27^. To limit the influence of remaining spurious sequences and possible nuclear-degenerated copies (*numts*), which are known to occur when working with mitochondrial DNA (e.g., Song et al.^28^), the program LULU^29^ was applied to the COI data, with an identity of 0.84 and a co-occurrence of 0.9. For analyses, the number of reads representing each OTU was then transformed as a percentage (relative to the total number of reads per sample for the target marker), in order to infer the evolution of the prevalence of DNA from each prey taxa along the survey.

## Results/discussion

Our results clearly illustrate that following a period of assisted feeding as part of the rehabilitatiopn process, Hvaldimir was feeding on wild prey, thus confirming suspicions based on observations of feeding habits made during the rehabilitative process (Table 1, Fig. 1). The very first feces sequences detected were assigned exclusively (sample #1) or almost so (sample #2) to a nematode (*Anisakis simplex*). In contrast, the last samples (samples #4 to 6) showed an alternation of herring (*Clupea harengus*) and saithe (*Pollachius virens*). The latter is a species native to the region and shows active foraging and predation by Hvaldimir, in line with the known predatory behavior of wild belugas who feed on pelagic and benthic fishes in the open sea as well as in coastal and fjord areas^30^. The near absence of fish detected in feces collected during the first period spent in Hammerfest, despite Hvaldimir already having been provisioned with herring at the time, indicates a low ingestion of fish and/or a sampling performed too long after the last “meal”. Nevertheless, the evolution of the diet fits the timeline of the feeding by NOS, as well as increases in foraging behaviour and an enlargement of his geographical range (as indicated by the survey with the datalogger and GoPro images), and confirms active hunting by Hvaldimir, at a minimum, from September.

**Figure 2:**
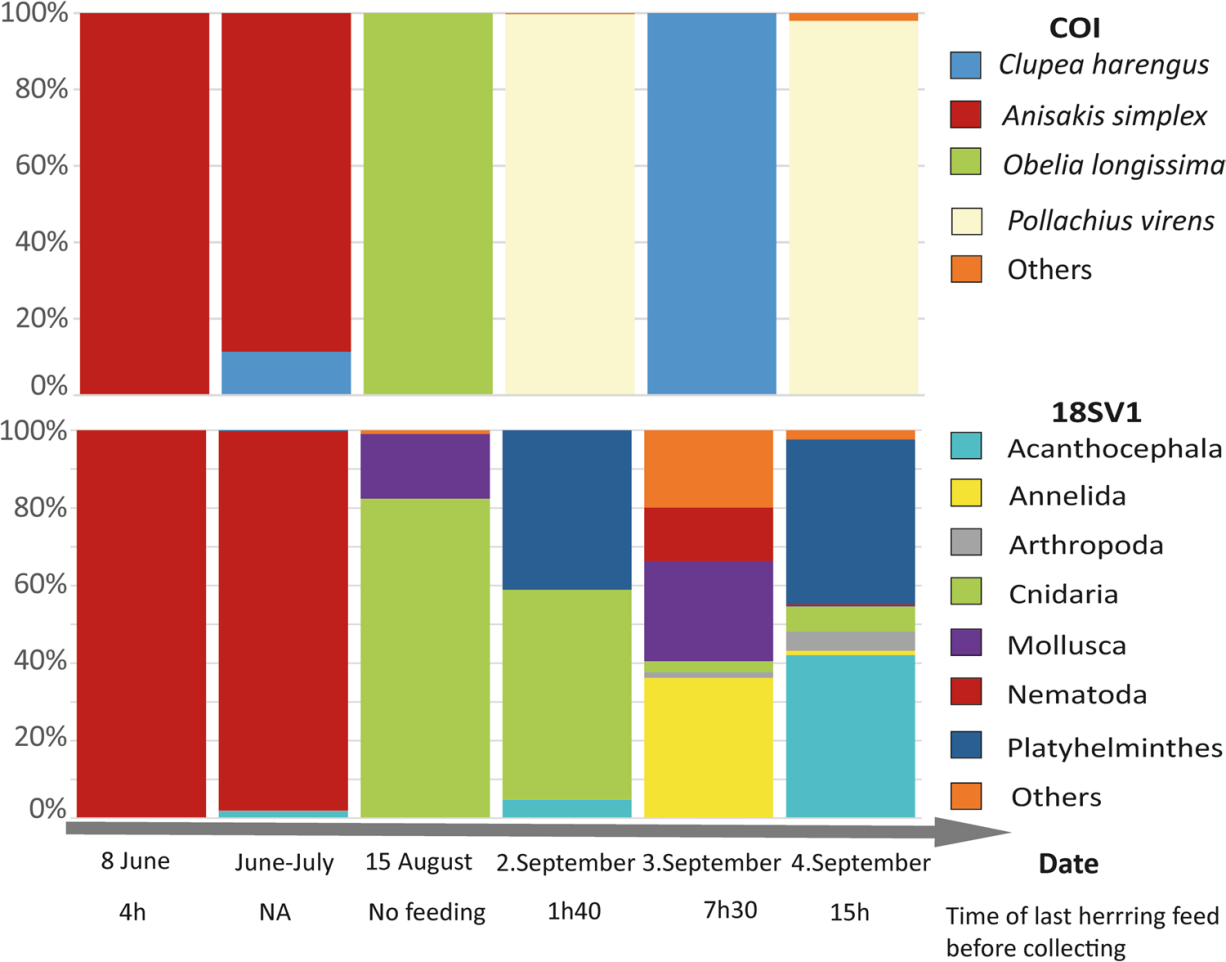
Relative proportions, generated separately for each gene region for each sample, of taxa encountered from COI (top) and 18S (bottom) metabarcodes, excluding all non-metazoan and beluga clusters. Time since last feeding is also detailed in Table 1.

Remarkably only saithe was identified on September 2nd (< 2 h after Hvaldimir was fed with herring) and 4th (15 h after he was fed with herring) with 94% and 99% of total reads assigned to this species. Contrastingly, on the day in-between (Sept 3th), 7.5 h after being fed with herring, only herring was detected (100% of total reads). This suggests the time of detection of preys after ingestion is higher than the digestion times previously reported for another delphinid, the harbour porpoise^31^, where a lag in detection was estimated to be about ~ 4.5 h, based on the passing of dyed fish. Our results suggest the period between feeding activity and defecation can exceed 7 h for belugas. In addition, below 2 h and after 15 h, no trace of herring was detected through dDNA, indicating a timeframe for detection that may guide future surveys or monitoring programs aiming to better reference daily diet as well as rhythmic changes in feeding (or other short periods).

Studies of diet composition from wild/dead beluga have typically relied on morphological stomach contents gathered from dead animals, stable isotopes, and fatty acid analyses^32,33^. The beluga whale is described as an opportunistic predator, targeting most commonly Atlantic cod as well as other fish and invertebrates, predominately shrimps, cephalopods, echiurids and amphipods^34,35^. Changes in diet composition occur frequently by region, depth, season and size of the beluga^32–34^. Although passive or secondary (ie preys of preys) predation may explain part of the invertebrates found in these inventories, results here are in line with previous morphological studies, and suggest a broader and more diverse range of prey are consumed, including many small or soft-bodied species^10^. This was most prominent during the second period of monitoring, when Hvaldimir enlarged his geographical range. Taxa detections using 18S-V1 included Acanthocephala, Annelida (Polychaeta), Arthropoda (Malacostraca, Maxillopoda), Ctenophora, Cnidarian (Hydrozoa, Myxozoa), Echinodermata (Ophiuroidea, Holothuroidea, Echinoidea), Gastrotricha, Mollusca (Bivalvia, Gastropoda), Nematoda, Platyhelminthes (Cestoda, Monogenea, Trematoda), Porifera (Calcarea, Demospongiae), Rotifera (Monogononta), Tunicata (Appendicularia, Ascidiacea). Long-term time-series of stomach contents through morphological inventories inventories support these observations, with reports that include Polychaeta, various Crustacea, Gastropods, Bivalves, and Cephalopods^32^.

While absolute biomass cannot be reliably estimated using metabarcoding approaches, several recent studies have shown that in comparison to other methods, relative read abundance can be used to estimate relative biomass for vertebrate as well as invertebrate feces/stomach content within one experiment^3,10,36^. Here, the high relative abundance of reads assigned to mussels (*Mytilida* sp. with up 26%) and polychaete worms (Polycheta with 36%) may thus reflect active predation. However, based on the size and relative abundance of other taxa, we could not discard contamination from surrounding water or secondary predation^3^ as alternative hypotheses for their detection. It is nevertheless interesting to point out a large relative abundance of reads for Cnidaria in August and early September. Both gene regions detect mostly cnidarian DNA, *Obelia longissimi* from COI and a large cluster of Hydrozoa and Myxoyoa from 18S. In this particular area, *Obelia* sp. is found in the saithe diet^37^, and this observation may thus either reflect opportunistic “grazing” or earlier undetected diversification through active hunting of saithe.

Among invertebrates, several detected OTUs were assigned to parasitic taxa, including Platyhelminthes (Trematoda, Cestoda) and *Echinorhynchus gadi*. These are known parasites of saithe, cod, herring, and others^38,39^, their detection is in line with the presence of saithe and herring in Hvaldimir’s feces. The detected parasitic nematode *Anisakis simplex* also known as ‘herring worm’, due to famous human infestation through the consumption of raw herring and capelin alongside other seafood^40–42^, has a life cycle pervasive across marine food webs, from mammals to fishes and arthropods. It is thus a common parasite of belugas, and known to be associated with natural prey species. The occurrences of nematodes in fish or marine mammals can nevertheless have health implications (for example, chronic ulcerative gastritis), and in extreme cases lead to death^43^, as implicated by many stranded porpoise across northwest Europe^44^. The overwhelming relative abundance of *A. simplex* in the first two samples (90–100% reads for both gene regions), might thus reflect an acute parasitosis, at the time when the body condition of Hvaldimir’ was showing serious signs of malnutrition. We cannot distinguish if the nematode was the reason for his poor condition, or if infested herring consumption in tandem with a weak body condition potentially favored the growth of the parasite population. Decreases in the relative abundance of *A. simplex* to < 1% in September (see Fig. 2), along with increases in diet diversity were supportive of Hvaldimir’s improved health over-time, as further confirmed by visual observations of improved body condition and energy levels (Fig. 1A–C).

Deagle et al.^3^ and others outline how relative read abundance information, using thresholds to limit common biases, provide precise detections in population-level diet assessments. Results obtained here support the application of this technique for the temporal assessment of individual-based read abundances, and as an indicator of hunting behavior and changing health status during rehabilitation programs.

## Conclusions

This short case study on the beluga whale ‘Hvaldimir’ demonstrates the power of dDNA analyses of feces as a non-invasive tool to monitor changes in diet and parasitic status of individual specimens, with particular relevance to cases of rewilding or the rehabilitation of animals that were once dependent on human care. In addition, results show that the time frame for feces collection is very important, and that detection may not occur until at least 7 h 30 min after feeding, which is much longer than that expected based on literature on other marine mammals species. A maximum detection window of around 15 h is recommended. These observations support the use of pilot studies to identify the proper time frames within which sample collection should occur, before setting up monitoring protocols. In addition, the results encourage the further investigation of the use of dDNA relative read abundance at an individual level to study marine mammal feeding behavior through time.

## Data Availability

The deposition of DNA sequences: raw sequences are available at the Sequence Read Archive (SRA): SRR15570209-SRR15570217, with BioSamples SAMN20927803-SAMN20927811, BioProject: PRJNA756855.
